# Foraging dives of southern right whales (*Eubalaena australis*) in relation to larger zooplankton size prey availability in Golfo Nuevo, Península Valdés, Argentina

**DOI:** 10.1038/s41598-024-63879-y

**Published:** 2024-06-20

**Authors:** Valeria C. D’Agostino, Ariadna C. Nocera, Kyler Abernathy, Alex Muñoz Wilson, Mariano A. Coscarella, Mariana Degrati

**Affiliations:** 1 Laboratorio de Mamíferos Marinos, Centro para el Estudio de Sistemas Marinos (CESIMAR), CCT CENPAT, CONICET, Blvd. Brown 2915, U9120ACV Puerto Madryn, Chubut Argentina; 2 Centro para el Estudio de Sistemas Marinos (CESIMAR), CCT CENPAT, CONICET, Blvd. Brown 2915, U9120ACV Puerto Madryn, Chubut Argentina; 3 National Geographic Exploration Technology Lab, Washington, DC 20036 USA; 4 Pristine Seas, National Geographic Society, 1145 17th Street NW, Washington, DC 20036 USA; 5 Universidad Nacional de la Patagonia, San Juan Bosco, Blvd. Brown 3150, U9120ACV Puerto Madryn, Chubut Argentina

**Keywords:** Ecology, Behavioural ecology

## Abstract

Southern right whales (SRWs, *Eubalaena australis*) have been observed feeding both at and below the surface (< 10 m) in Golfo Nuevo (42°42′ S, 64°30′ W), Península Valdés, Argentina, an area traditionally recognized as calving ground. In addition, we documented diving feeding behavior in SRWs during their stay in this gulf, which has not been previously described. We assessed this behavior using suction-cup-attached video-imaging tags (CRITTERCAMs) on individual whales. A total of eight CRITTERCAM deployments were successful, and feeding events were documented in all SRWs successfully equipped with CRITTERCAMs. The highest speeds occurred during the ascent phase, and the average diving time was 6 min 45 s ± 3 min 41 s for SRWs. Concurrently, zooplankton samples were collected from the subsurface and bottom of the water in areas where tagged whales dived to assess differences in composition, abundance, and biomass. Copepods dominated the upper layer, while euphausiids were more abundant in the deeper sample. Furthermore, zooplankton total biomass was five times higher at depth (2515.93 mg/m^3^) compared to the subsurface (500.35 mg/m^3^). Differences in zooplankton characteristics between depths, combined with CRITTERCAM videos, indicated that SRWs exploit high concentrations of organisms near the seafloor during daytime feeding dives. This study provides baseline insights into how SRWs utilize Península Valdés during their stay in the area.

## Introduction

Studying the behavior of marine mammals poses many challenges due to their prolonged periods spent underwater. In most cetaceans and other marine mammal species, foraging and feeding take place below the water’s surface^1^, making direct observation of these behaviors from the surface impossible. Consequently, most conclusions regarding the diets of marine mammals have been derived from molecular methods, such as stable isotope and fatty acid analyses (e.g.^2–4^). These conclusions have also been drawn from opportunistic sampling of gastrointestinal tracts (stomach and intestine) and feces from both stranded and live cetaceans and pinnipeds (e.g.^5–8^), as well as from both directed catches and accidental captures (e.g.^9,10^). Only a few studies have described the dietary composition of marine mammal species through to observations of their feeding habits (e.g.^11–14^). Additionally, while surface photographs and video clips from aerial drones or underwater cameras have successfully captured behaviors at or just below the water surface in marine mammals (e.g.^15–17^), these images are unable to document their deep-sea behavior. Fortunately, progress in imaging technology over the last three decades has led to the development of small, minimally invasive devices for animal-borne video, audio, and data-logging deployments, enabling thorough documentation of their behavior^18,19^. The miniaturization of video technology has allowed researchers to observe the marine habitat from the animal’s perspective and record their behavior in the deep oceans^18–22^, becoming a powerful tool for studies on marine mammals.

The southern right whale (SRWs, *Eubalaena australis*) is one of the three baleen whale species in the genus *Eubalaena*. It is a large mysticete, weighing up to 60 tons with a body length between 13–16 m, and has a uniquely shaped head comprising about one-quarter to one-third of the total body length^23^. The SRW has a circumpolar distribution in the Southern Hemisphere between 12°S and 65°S^24^, migrating annually between productive feeding and sheltered calving grounds. SRWs are filter-feeders, using their baleen to filter prey from dense patches of zooplankton (mainly copepods and euphausiids)^9,17,25^. Feeding typically occurs in austral summer and fall in regions located around 40°S and 65°S^26–29^. By late fall, SRWs leave their feeding grounds to mate, give birth, and nurse their calves in calving grounds situated near coastlines from about 27°S to 50°S^26,27,30^. After being extensively hunted by commercial whaling from the 18th through the early twentieth centuries, the main breeding populations (Argentina/Brazil, South Africa, Australia and New Zealand) of SRWs have shown evidence of strong recovery^24,31–33^. The global SRW population is estimated to have increased at a rate of approximately 7% per annum, reaching 13,600 individuals in 2009^34^. The total population size for the SRW in the Western South Atlantic Ocean has recently been appraised at 4,742 individuals^33^. The species is globally classified as “least concern”^35,36^.

Copepods and euphausiids occupy a key position in the pelagic food webs, feeding on phytoplankton, small heterotrophic organisms, and detritus^37,38^. Additionally, they serve as a significant food source for fish, larger zooplankton organisms, and some marine mammals, including right whales^7,13,38–40^. Many large, energy-rich copepods and euphausiid species change their vertical distribution in the water column in a daily cycle (diel vertical migration DVM)^41^. This behavior involves a vertical migration to deeper waters before dawn and an upward migration at dusk towards the surface layer^41,42^. During DVM, zooplankton migrate to the surface to feed at night and then return to deeper depths to reduce predation risk from visual predators such as zooplanktivorous fish^43–45^. Studies have shown that larger zooplankton in advanced developmental stages, such as late juveniles and adults, tend to undergo DVM to greater depths compared to smaller individuals in earlier developmental stages^46–48^. While evasion of predators is the most likely benefit^41^, several hypotheses explain this behavior, including the response to light intensity, taking advantage of the metabolic benefits of living in colder depths during the day, and cost-effective feeding^49^. This synchronized movement of zooplankton can generate dense aggregations of organisms near the seafloor, concentrating them in both space and time, thereby making them more accessible to zooplanktivorous animals.

**Figure 1:**
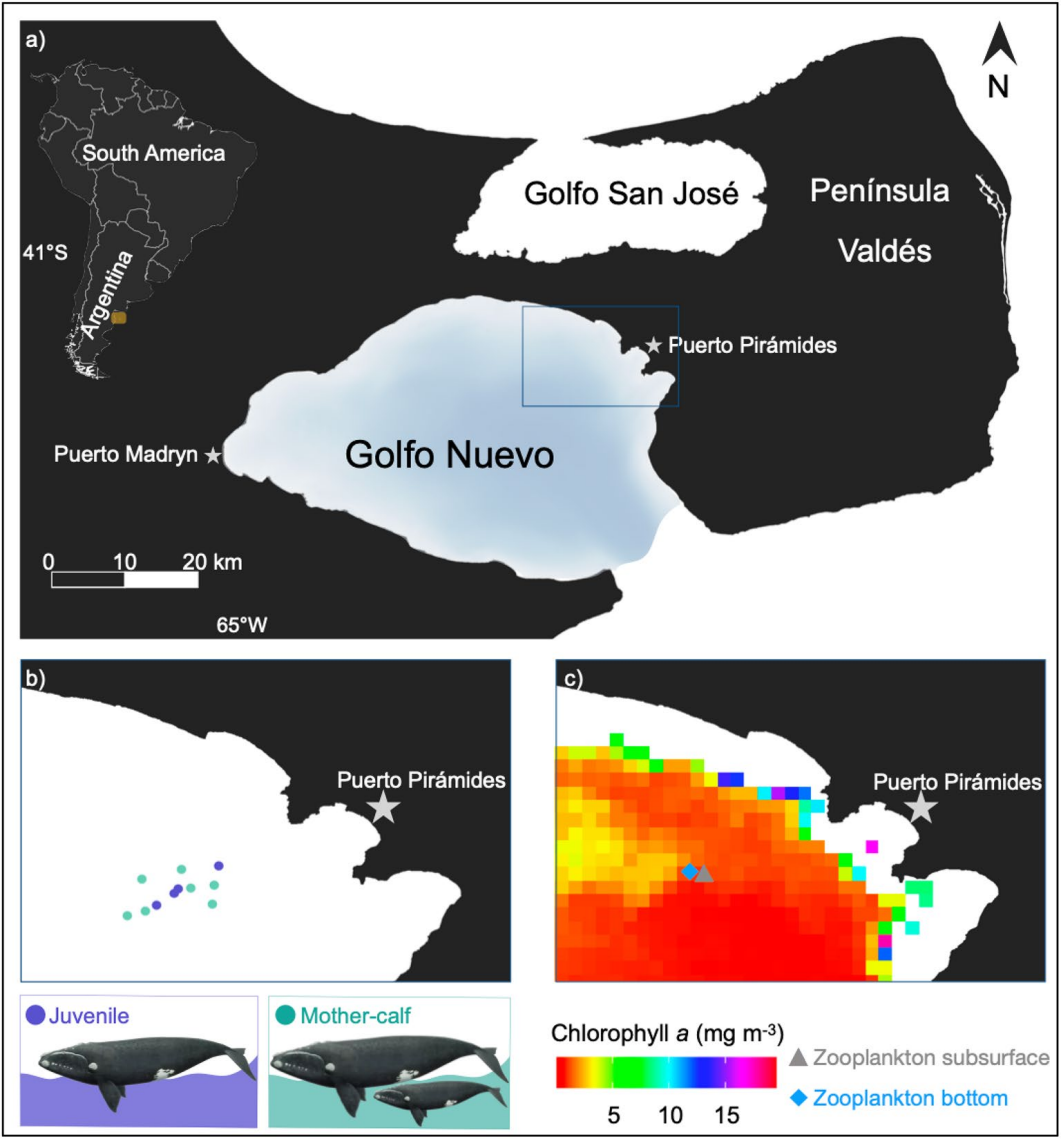
(**a**) Map of the study area showing the locations of CRITTERCAM deployments (blue rectangle) on southern right whales (*Eubalaena australis*), (**b**) zoom to the individuals positions (juveniles and mother-calf) and (c) zoom to the sampling area for the zooplankton net tows at the subsurface and bottom (30 and 100 m, gray triangle and blue rhombus, respectively) in Golfo Nuevo, Patagonia, Argentina (created in QGIS 3.4.7-Madeira). Background in (**c**) represents monthly average satellite chlorophyll *a* (mg m^−3^) during October 2022 (https://www.oceancolor.gsfc.nasa.gov).

The gulfs bordering Península Valdés—Golfo San José to the north and Golfo Nuevo to the south (Fig. 1)—are important calving and mating grounds for the SRW population in the western South Atlantic Ocean. It has been estimated that close to 36% of the SRW population visits the calving ground off Península Valdés, Argentina, every year^33^. Various aspects of SRW ecology and biology at Península Valdés, including population dynamics, movements, behavior, and reproduction, have been periodically studied^7,13,29,50–53^. Additionally, although it was previously believed that SRWs feed only in feeding grounds and not in calving grounds such as Península Valdés^54^, investigations have demonstrated that SRWs do feed in the gulfs off Península Valdés^7,13,55^. Furthermore, a recent study identified the waters off Península Valdés as a multi-use habitat for SRWs^17^. An analysis of photographic and video data from 2007 to 2019 conducted by D’Agostino et al.^17^ revealed that SRWs feed annually throughout the calving season at Península Valdés (June to December), primarily during the spring months. Studies have demonstrated that at Península Valdés, SRWs mainly feed on adult and fifth copepodite (CV) stage of calanoid copepods (*Calanoides carinatus*, *Calanus australis*, *Ctenocalanus vanus*, and *Paracalanus parvus*), zoeae of squat lobster (*Grimothea gregaria*), calyptopis and furcilia of euphausiids (*Euphausia lucens*), as well as fish eggs and larvae^7,13,55^. D’Agostino et al.^17^ observed SRWs feeding both at and below the surface (< 10 m) and suggested that SRWs dive to feed near the bottom, evidenced by observations of individuals surfacing with mud on their heads. However, to our knowledge, conclusive evidence of SRWs feeding near the bottom in Península Valdés is lacking. Moreover, the behavior of SRWs during deep foraging and their potential prey remains unknown. This study aimed to characterize the diving behavior of SRWs during the austral spring in Golfo Nuevo and infer its potential functions using suction-cup-attached video-imaging tags (CRITTERCAMs) on SRW individuals for the first time. Additionally, we collected zooplankton samples to explore differences in community composition and abundance between the subsurface and bottom when SRWs dive in Golfo Nuevo. The present results introduce a novel approach in the region to investigate SRWs and emphasize the importance of understanding their behavior and habitat use for implementing effective protection measures. In addition, to date, there is no data on the species composition and abundance of the zooplankton community near the bottom in Golfo Nuevo. Hence, this study will provide context for future research on the vertical distribution of zooplankton in relation to the behavior of zooplanktivorous animals within the Península Valdés area.

## Materials and methods

### Study area

Golfo Nuevo, located in the Península Valdés region of Argentina (42° 42’ S, 64° 30’ W, Fig. 1), is a semi-enclosed basin that covers approximately 2400 km^2^. It has an average depth of 80 m and reaches a maximum depth of 180 m^56^. The gulf is connected to the Southwestern Atlantic through a 16-km-wide gap facing southeast (Fig. 1). As a result, the water’s dynamics in Golfo Nuevo are primarily influenced by atmospheric forces rather than those of the adjacent shelf^56,57^. Fieldwork was conducted at a specific site within Golfo Nuevo known as “El Nido” (Fig. 1), where it is common to observe SRW individuals spending extended periods diving and often resurfacing with mud on their heads during daylight, especially from mid to late spring^17^. The depth at El Nido varies between 80 and 140 m.

### Permits, ethic statement and approval

CRITTERCAM field procedures were conducted in accordance with the relevant guidelines and regulations imposed by the Dirección de Fauna y Flora Silvestre and Subsecretaría de Conservación y Áreas Protegidas of Chubut Province, Argentina under sampling permits n: 84/2022 and 85/2022. The research permits also included the necessary ethical approval in terms of sample collection, analysis and use for scientific studies.

### CRITTERCAM deployments

**Figure 2:**
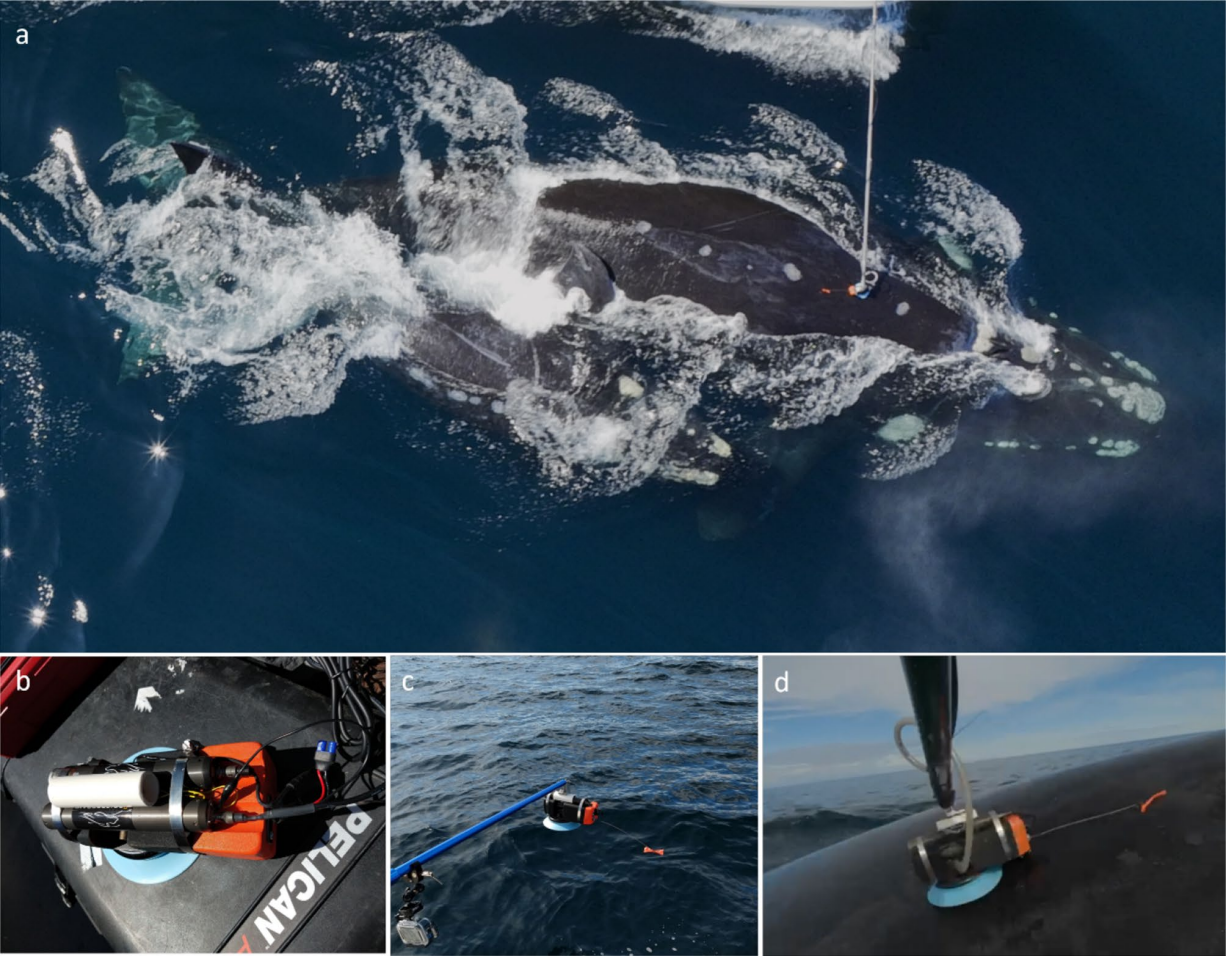
(**a**) CRITTERCAM deployments on southern right whales (*Eubalaena australis*) in Golfo Nuevo, (**b**) CRITTERCAM system, (**c**) CRITTERCAM system attached to deployment pole, (**d**) CRITTERCAM deployed on an individual southern right whale.

In collaboration with the National Geographic Exploration Technology Lab and National Geographic Pristine Seas Expeditions, we deployed the CRITTERCAM in Golfo Nuevo between October 4^th^ and October 31^st^, 2022 (Table 1, Fig. 2a). Deployments were exclusively carried out during daylight hours. The CRITTERCAM collected high-resolution video (1280 × 720 pixels, 30 frames s^−1^) and included an onboard sound recorder, as well Star-Oddi DST milli-F loggers which were set to record temperature and depth every 1 s. The recording camera was housed in a 20-cm-long × 3.2-cm-diameter aluminum cylinder, and paired with a second housing of the same design that provided light from high-output LEDs. The full CRITTERCAM system (including CRITTERCAM, light, suction cup and polyurethan foam for floatation) measured approximately 30 cm in length, 10 cm in height, and 7.6 cm in width (widest part except for the suction cup) (Fig. 2b). CRITTERCAM system weighed approximately 1.1 kg in air and was slightly positively buoyant in water. It was equipped with a VHF transmitter for tracking and retrieval once released from SRW.

**Table 1:** CRITTERCAM deployments on southern right whales (*Eubalaena australis*) in Golfo Nuevo, Península Valdés.

ID	Date	Age class	Deployment duration (h:min:s)	Site depth (m)	Max depth (m)	Max dive duration (min:s)	Dive descriptions	Prey observation
1	10/4/23	Adult female	01:23:03	~ 110	~ 112	02:37	Feeding subsurface (< 10 m) and dive. Calf visible at ~ 100 m. 2nd whale visible feeding at ~ 3 m (Video S2)	Presumably zooplankton prey visible from ~ 10 m to deep
2	10/5/23	Adult female	00:12:25	~ 94	~ 84	01:56	Feeding dive. Calf visible alongside its mother during the descent at ~ 84 m (Video S3)	Presumably zooplankton prey visible from ~ 25 m to deep
3	10/5/23	Adult female	00:02:31	~ 120	–	–	Unsuccessful deployment (< 10 min)	–
4	10/5/23	Juvenile	02:19:54	~ 132	~ 108	12:48	Feeding dives at two depths, ~ 30 to 40 m and ~ 100 m (Video S4aand b, respectively)	Presumably zooplankton prey visible from ~ 28 m to deep
5	10/5/23	Adult female	03:07:24	~ 106	~ 19	04:38	Most of time in subsurface (< 10 m). One dive at ~ 19m	Little amount of presumably prey visible at ~ 15 m
6	10/7/23	Adult female	00:38:00	~ 100	~ 60	–	No video. Only depth data	–
7	10/7/23	Juvenile	00:02:35	~ 110	–	–	Unsuccessful deployment (< 10 min)	–
8	10/7/23	Juvenile	00:45:41	~ 118	~ 115	04:48	Feeding dive (Video S5)	Presumably zooplankton prey visible from ~ 30 m to deep
9	10/9/23	Adult female	00:30:14	~ 123	~ 5	–	Subsurface (< 5 m)	Presumably zooplankton prey visible at ~ 5 m
10	10/11/23	Adult female	00:25:19	~ 76	~ 75	12:07	Feeding dive. Calf visible at ~ 73 m (Video S6)	Presumably zooplankton prey visible from ~ 48 m to deep
11	10/31/23	Juvenile	lost camera	~ 92	–	–	–	–
12	10/31/23	Adult female	lost camera	~ 123	–	–	–	–

*ID* deployment number, –, data not available.

CRITTERCAM deployments were conducted by approaching whales in a 7.5 m rigid-hulled inflatable boat at a range of approximately 1–5 m. The CRITTERCAM was deployed using a 3–6 m pole and attached to the SRWs with a silicone suction cup of 18 cm in diameter, using a remote vacuum pump to generate active suction between the SRW’s skin and the suction cup (Fig. 2a, c, d). CRITTERCAMs were mounted on the dorsal body area of the whales, just behind the blowholes (Supplementary Video S1 online, Fig. 2a, d). In this position, it provided a forward-looking view of the whales and their surroundings. The system initiated recording immediately upon deployment, triggered by an immersion sensor, and continued until either the video memory reached capacity (ten hours of maximum) or the pre-programmed release time of day. After the CRITTERCAM was attached, we registered the age class and/or sex of tagged SRW, its position with a GPS, the depth of the site where the whale was tagged using an echosounder and took photographs of the individual. Sex was determined for adult females by observing whales closely accompanied by a calf. However, the sex of juveniles was not determined because it was not possible to observe the shape of the genital area^58^. Juveniles were identified by their evidently smaller size compared to adult whales^58^.

Once the CRITTERCAM was released from the SRW (either at a scheduled time, upon completing recording time, or due to the whale’s activities), the device floated to the surface and was recovered by a boat-based recovery team. Successful deployments were defined as those with a tag duration greater than 10 min and full tag and data recovery. The data from the deployments were downloaded and analyzed using the Crittercam MultiMode programming interface. SRW dives were defined as any vertical descent below 10 m, whereas movements of whales between 0 and 10 m were considered within the subsurface layer^17^. The descent and ascent rates of the dive were calculated by dividing the distance traveled during each phase by the respective time durations. Here, we calculated the dive rates through the diving profile of whales that travelled directly to the seafloor and returned to the surface. Dives were classified into 2 types: V-shaped and U-shaped based on dive profiles^59^. Feeding dives were identified based on visual assessment of video footage, which involved observing increased particle density (likely zooplankton) and accelerated particulates flowing past in the video, along with observations, when feasible, of the SRW’ heads moving upward and downward, indicating potential mouth opening during swimming.

### Zooplankton sampling and analysis

Zooplankton samples were collected on October 31^st^, 2022, by net tow during daylight hours from both the subsurface (~ 30 m)^60^ and the bottom (~ 100 m) at the location where all SRWs tagged in this study were recorded diving (Fig. 1). Zooplankton subsurface sample was collected using a 333 μm plankton net, with a 50 cm mouth diameter equipped with a mechanical General Oceanics flowmeter (model 2030R) on the net mouth. For the bottom-depth tow, we designed a protective, heavy sled to ensure the net reached the seafloor and captured the zooplanktonic organisms aggregated near the bottom, while preventing damage to the net during the seafloor tow (Supplementary Fig. S1 online). The sampling system kept the net positioned 15 cm above the seafloor. The bottom tow was performed using the sled equipped with a plankton net (335 μm mesh, 50 cm mouth diameter) with a mechanical General Oceanics flowmeter (model 2030R) on the net mouth and sensors of temperature and pressure. These mesh sizes were chosen to replicate the capture of zooplanktonic organisms by the right whales’ baleen, according to Mayo et al.^61^. Both subsurface and bottom samples were collected through a horizontal net tow for a period of 10 min from a motor boat at ∼2 knots forward speed. The samples were stored in 500 mL (subsurface) and 1000 mL (bottom) plastic flasks and preserved with 4% formaldehyde for later analysis. The bottom net did not have a closing mechanism, so some sampling overlap may have occurred when bringing the 100 m tow back to the surface along with the 30 m sample. However, because this process follows a vertical path and takes only a short amount of time, the overlapping volume is minimal compared to the total filtered volume (i.e., 617.33 m of horizontal sampling during the 10-min boat excursion). It is important to note that the flowmeter readings were zero during the descent^60^. Therefore, considering the differences in distance, time, and volume between boat and manual sampling, we do not expect significant errors in our data from this overlap. Consequently, the representation of the bottom zooplankton community is reliable. Zooplankton samples were examined under a S8 APO Stereozoom 1.0×–8.0× Leica stereoscope for enumeration and taxonomic analyses. Samples were divided in aliquots (1/10 sample volume) after homogenization^62^ and all individuals in a subsample were identified to the lowest taxonomic level possible using appropriate literature^62–64^. The adults of calanoid copepod species found in the samples, namely *P. parvus*, *C. vanus*, and *C. australis* (Supplementary Fig. S2 online), as well as CV males of *C. vanus*, were all sexed based on clear morphological features. Early copepodite CI–CII–CIII–CIV and small CV were grouped without species or sex differentiation and categorized into either small (up to 1.8 mm^38^) or large (3.50 mm^38^) sized calanoid copepodites. The prosome length was measured for 30 randomly chosen adult female of *C. australis* from each sample depth (subsurface and bottom) using a S8 APO Stereozoom 1.0×–8.0× Leica stereoscope equipped with a MShot MSX1 Microscope camera. The measurements were exclusively conducted on adult females of *C. australis*, which were identified as the most abundant large calanoids in both depths. Differences (*p* < 0.05) between body size (prosome length) of *C. australis* females from the subsurface and the bottom were tested with the Mann–Whitney U test using R Statistical Software^65^ and the figure was generated using *ggplot2*^66^ package in R Statistical Software^65^. In addition, to estimate the biomass (wet weight) of copepod species, published individual length^38^ and a length–weight relationship were used^67^.

Euphausiids were identified up to species level and development stages. To estimate euphausiid biomass (the relationship between wet weight and subtotal length^68^), 10 furcilia IV-V (FIV-FV) and 30 juveniles of *Euphausia lucens* (Supplementary Fig. S3 online) were randomly taken from the preserved bottom sample and their subtotal lengths were measured (taken from the tip of the rostrum to the posterior end of the sixth abdominal somite^69^) using a S8 APO Stereozoom 1.0×–8.0× Leica stereoscope equipped with a MShot MSX1 Microscope camera.

Zooplankton abundances were expressed as the number of individuals per cubic meter (ind m^−3^). To test for significant differences (*p* < 0.05) in zooplankton abundances between depths (subsurface and bottom) Kruskal–Wallis test was performed, using the *vegan*^70^ package in R Statistical Software^65^. In addition, the Shannon–Weaver index (H’) was calculated^71^, based on the species composition and their abundances to estimate the zooplankton community diversity for the two depths and the significance between these indices was assessed performing the Hutcheson’s t-test using *ecolTest*^72^ package in R^65^. Furthermore, through direct observation of the CRITTERCAM video footage, the variation in prey abundance with depth could be identified.

## Results

### CRITTERCAMs data

#### Environmental variables

The temperature in the water column was on average 10.71 ± 0.47 °C during all the registered dives by SWRs in Golfo Nuevo, with a higher mean of 10.81 ± 0.4 °C in the upper 40 m and 10.05 ± 0.3 °C in depths greater than that depth (17.56 and 9.59 °C, were the maximum and minimum recorded, respectively). No thermocline was evident from these profiles. In the case of the euphotic layer, this could be visually assessed as the depth where the videos lost the illumination and was found at around 41.56 ± 3.83 m.

#### Deployment and recovery

Of the 12 CRITTERCAMs deployments on SRWs in Golfo Nuevo during October 2022, only 8 were considered successful deployments (Table 1). In one case, a technical problem resulted in no video recorded; however, the system registered depth data (ID6). ID7 deployment was considered unsuccessful since its duration was less than 10 min. In two other deployments (ID11 and ID12) the loss of CRITTERCAM led to no data recovery (Table 1). In both cases, the CRITTERCAMs were deployed correctly, but unfortunately, they were not retrieved. The duration of deployment varied greatly, often as a result of the activities of the SRW individuals which led to removing the CRITTERCAM. The deployments lasted on average 58 min 47 s ± 66 min 02 s (range 12 min 25 s–187 min 24 s).

#### Dive behavior observations

**Figure 3:**
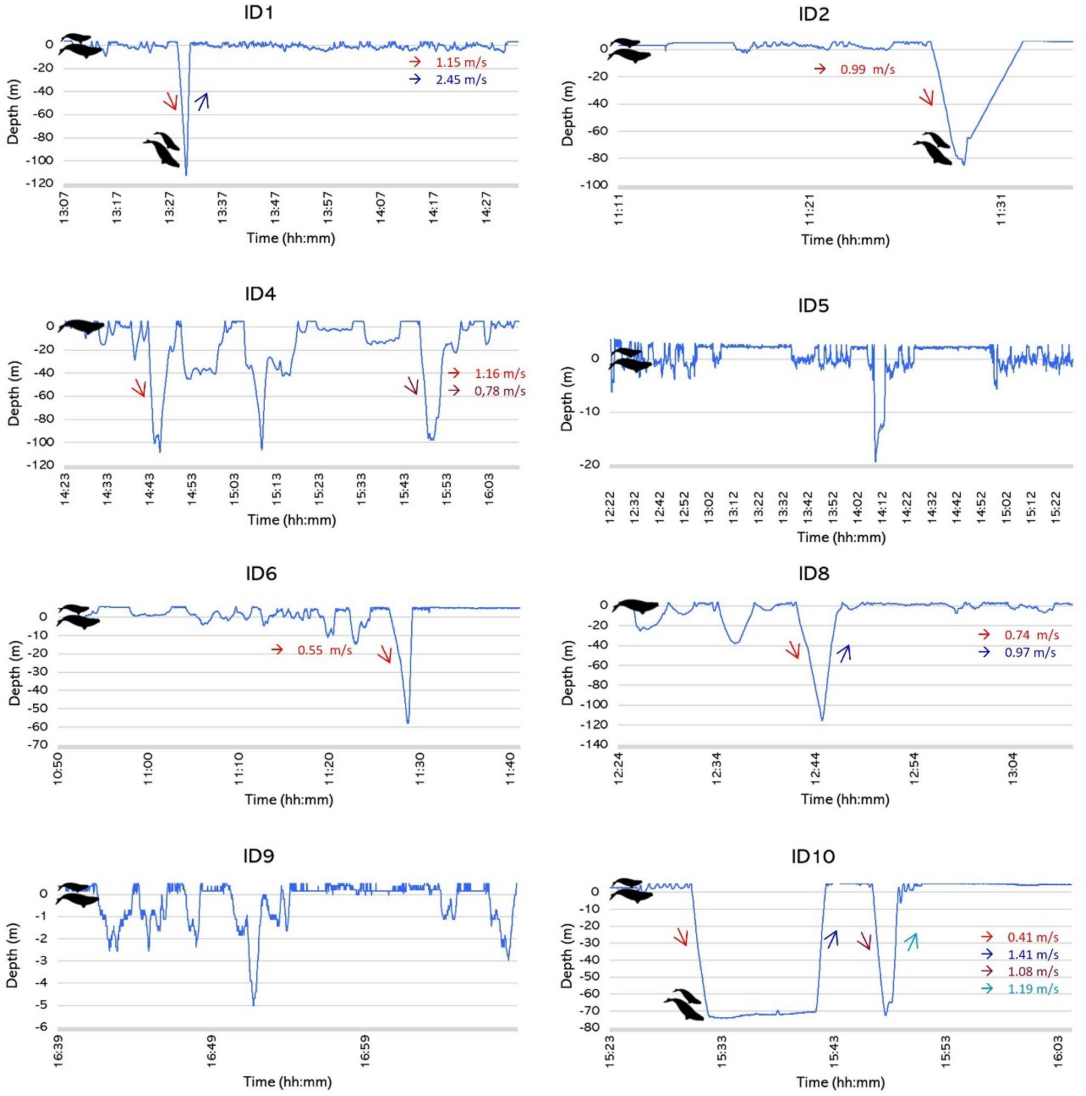
Dive profiles of southern right whales (*Eubalaena australis*) in Golfo Nuevo observed using CRITTERCAMs. Including vertical speed (m/s) of descent and ascent (when calculation was possible). ID: deployment number. Schematic black figures represent adult female (mother-calf pair) or juvenile individuals. Profiles ID1, ID2 and ID10 show the presence of a calf at depth. ID6 shows only descent speed due CRITTERCAM failed to collect video data; consequently, we were unable to determine how the camera reached the surface.

Of the total of 8 success deployments conducted, 5 were in adult females closely accompanied by their calves (mother-calf pair) and 3 juveniles of unknown sex (Table 1, Fig. 3). Only in deployment ID9 (adult female), the individual remained at the subsurface area (< 10 m), while all other tagged SRWs dove (> 10 m) (Fig. 3). Dive depths varied among individuals, ranging from shallow to the deepest possible given the water depth, with a maximum recorded depth of 115 m (ID8) (Table 1, Fig. 3). The lighting module on the CRITTERCAM was not powerful enough to make the seafloor visible from the tag’s location on the whales’ backs. However, because the depth recorded by the tags closely matched the documented bottom depths of the deployment site, we inferred that the whales reached the bottom during their deepest dives (Table 1). In addition, the lack of an accelerometer and magnetometer sensors in the CRITTERCAMs used did not allow us to determine the orientation of the whales.

The average diving time recorded was 6 min 45 s ± 3 min 41 s (minimum = 2 min 21 s; maximum = 12 min 48 s) (Table 1). The maximum number of dives recorded for one whale was five (ID4, Fig. 3). In addition, CRITTERCAM also provided data on ascent and descent rates. When the calculation of both speed of descent and ascent was possible, we observed that highest speeds were recorded during the ascent phase (Fig. 3). Of the eight dives recorded, seven were V-shaped, characterized by rapid descent and ascent with very little or no time spent at the bottom layer during the dive, while one was U-shaped, with the whale spent time near the sea floor during the descent and ascent phase (Fig. 3).

In three out of the five deployments on adult females (ID1, ID2, ID10), the videos also allowed us to observe calves’ dives. During these observations, the calves were registered in close proximity to their mothers at depth (ID1: 100 m, ID2: 84 m, ID10: 73 m; Table 1, Fig. 3). However, the position of the CRITTERCAM on the mother did not provide information about the activities of the calves at the bottom. Nonetheless, during deployment ID2, we observed the calf near its mother during the descent phase and at the bottom when the mother reached her maximum depth of 84 m (Table 1, Supplementary video S3 online).

Feeding events were documented in all SRWs successfully equipped with CRITTERCAMs, occurring during both deep and shallow dives. In the only case where the whale remained within the upper 10 m (ID9, Fig. 3), we inferred subsurface feeding behavior^17^ based on head movements and the occurrence of possible prey observed. Particularly in deployment ID1, the female (with CRITTERCAM attached) was initially observed filter feeding in the subsurface alongside another SRW individual (~ 3 m depth) and then the female was registered feeding at the bottom layer (Table 1, Supplementary Video S2 online).

### Abundance, composition and vertical distribution of zooplankton

**Figure 4:**
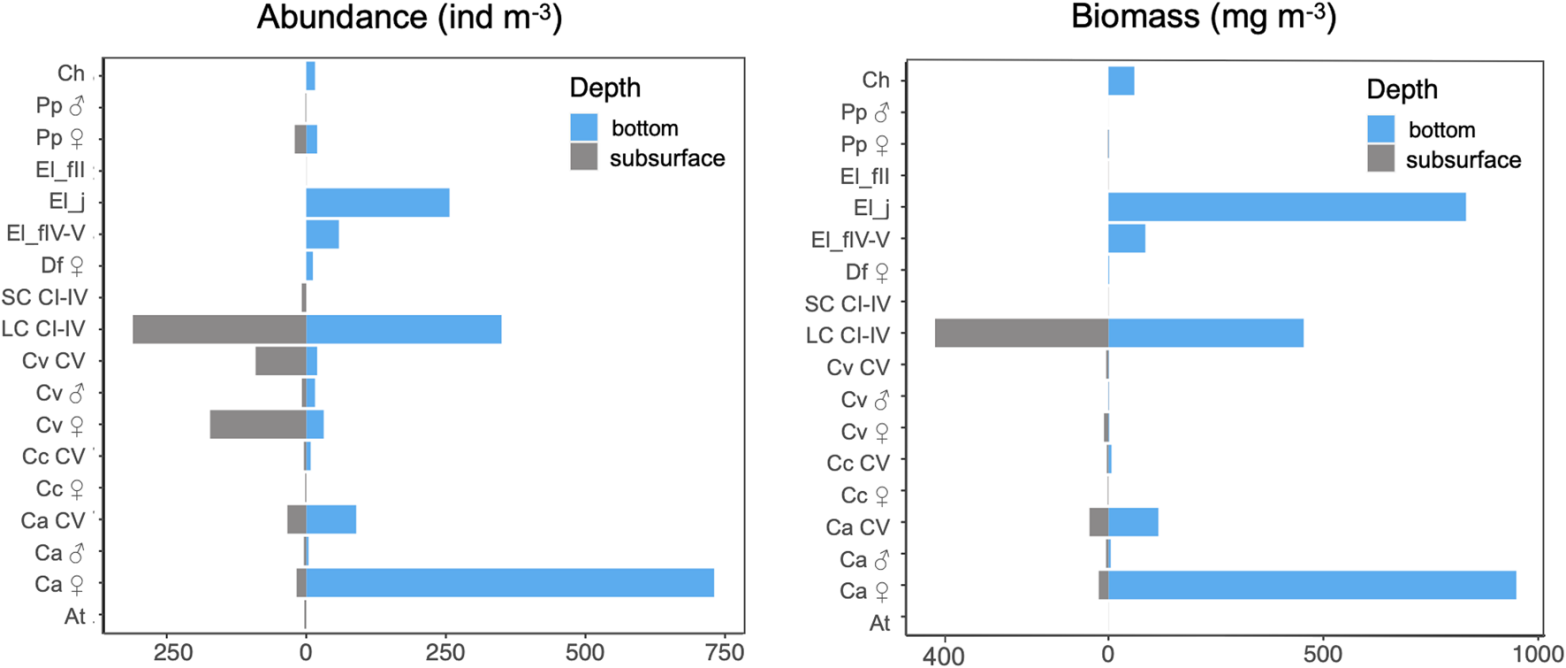
Abundance and biomass of zooplanktonic groups from the subsurface and bottom waters from Golfo Nuevo. Species/group codes: At, *Acartia tonsa*; Ca, *Calanus australis*; Cc, *Calanoides carinatus*; Ch, chaetognaths; Cv, *Ctenocalanus vanus*; CV, copepodites V; Df, *Drepanopus forcipatus*; LC I-IV, large calanoid copepodites (I–IV); SC I-IV, small calanoid copepodites (I–IV); El_f, *Euphausia lucens* furcilia II/IV–V; El_j *Euphausia lucens* juvenile; Pp, *Paracalanus parvus*. ♀: female, ♂: male.

**Figure 5:**
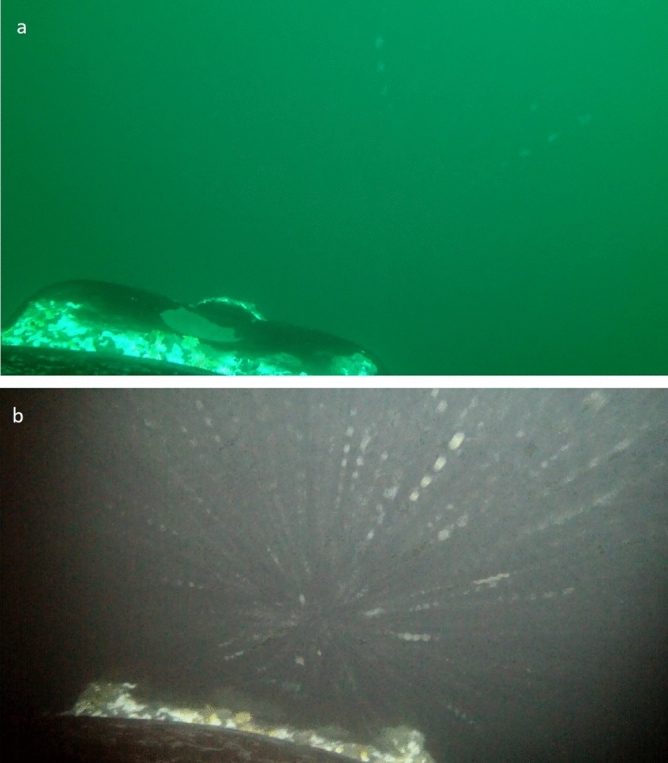
Images from CRITTERCAM on southern right whale, *Eubalaena australis*, (ID10). (**a**) During descent phase (~ 30 m) and (**b**) when whale feeding at bottom (~ 73) in Golfo Nuevo.

Total zooplankton abundances were significantly different (*p* < 0.05) between the sampled depths. Zooplankton were more abundant at the bottom compared to the subsurface (Fig. 4). The zooplankton abundance was 677.5 ind m^−3^ in the subsurface and 1608.6 ind m^−3^ in the bottom layer. Significant differences were further found for zooplankton species composition between the subsurface and the bottom (Hutcheson t-statistic = 2.91, df = 1294.3, *p* < 0.05). The Shannon–Weaver index (H’) indicated higher taxa diversity at the bottom (H’ = 1.28) compared to the subsurface (H’ = 1.16). At the subsurface, the zooplankton community was represented almost exclusively by copepods, reaching 99.96% of the total abundance of the organisms found (Fig. 4). The most abundant taxa were the large copepodites calanidae of *C. australis* and *C. carinatus* (45.85%), primarily dominated by smaller stages (CI–CIII), followed by adult females of the small copepod *C. vanus* (25.43%) (Fig. 4). At the bottom, the zooplankton community was dominated by adult females of the large copepod *C. australis* (45.41%). This species was followed by the large copepodites, mostly represented by CIV of *C. australis* and *C. carinatus* (21.74%). Copepodite V of *C. australis* was another numerically important component of the population of this copepod species at the bottom layer (5.56%) (Fig. 4). Euphausiids were more abundant at the bottom than in the subsurface (19.57% and 0.04%, respectively) (Fig. 4). In fact, this group was the second most abundant at the bottom and was represented exclusively by *E. lucens* stages juveniles (15.94%) and stages furcilia IV and V (3.62%) while at the subsurface, only furcilia II was present (0.04%) (Fig. 4). The total biomass of zooplankton (wet weight) was higher at the bottom (2515.93 mg m^−3^) compared to the subsurface (500.35 mg m^−3^). Adult females of *C. australis* and euphausiids (furcilias and juveniles) constituted the major fraction of the zooplankton biomass at the bottom, with similar values (948.91 and 917.60 mg m^−3^, respectively) (Fig. 4). Furthermore, the analysis of CRITTERCAM video images of diving whales revealed denser prey patches at greater depths in comparison to the subsurface (Fig. 5).

**Figure 6:**
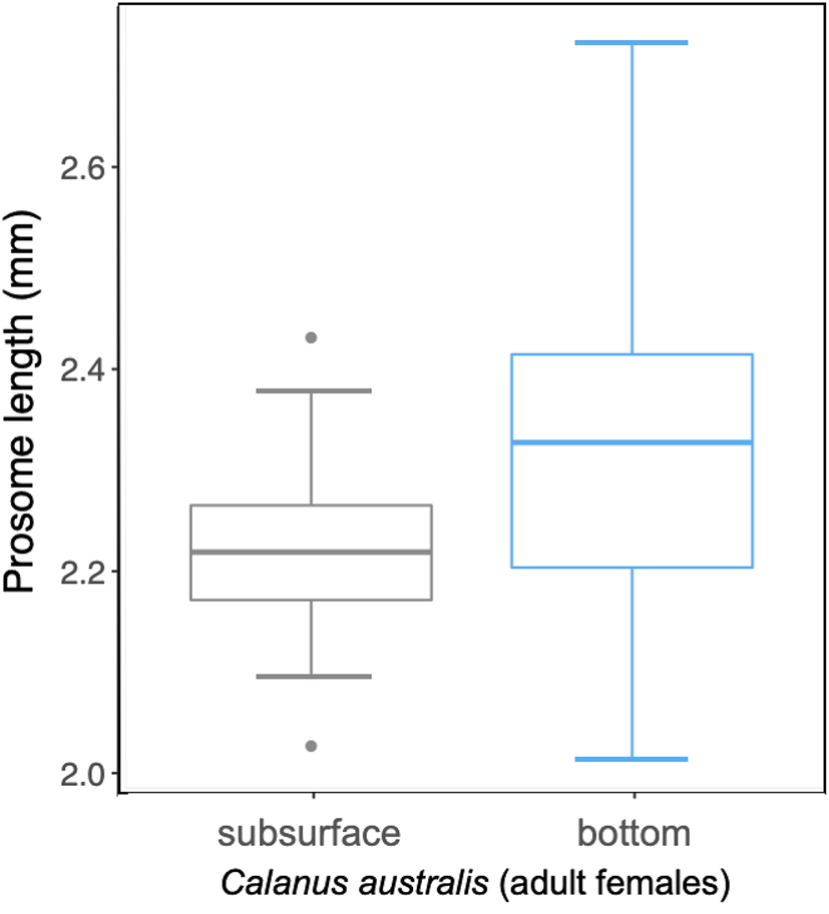
Boxplots display the mean (solid line in each box) and quartile ranges of the body size (prosome length) for *Calanus australis* adult females from the subsurface (left) and bottom (right) samples of Golfo Nuevo. Gray dots represent outliers found within the subsurface sample.

Significant differences were found for body size (prosome length) of adult females of *C. australis* between subsurface and bottom layers (*p* < 0.05). Mean values of 2.22 ± 0.09 mm (Fig. 6; range 2.03–2.43 mm) were found on the subsurface, while specimens from the bottom exhibited mean values of 2.33 ± 0.17 mm (Fig. 6; range 2.01–2.72 mm).

## Discussion

Our results provide the first strong evidence that SRWs dive into the deeper waters of Golfo Nuevo, Península Valdés, to feed on energy-rich zooplanktonic organisms. The observation of SRWs’ diving feeding at the bottom of Golfo Nuevo is in agreement with the feeding diving behavior described for North Atlantic Right Whales (NARW, *E. glacialis*) when they feed on larger-bodied calanoid copepod patches during summer feeding season in deep waters of the lower Bay of Fundy and Roseway Basin^59^. The present study revealed significantly higher abundance and biomass of large calanoid copepods and euphausiids in deeper waters compared to the subsurface within the same location. The older stage of large calanoid copepods (CIV–CV and adults) and euphausiids (FIV–FV and juveniles) are a high-quality food source because they accumulate large energy-rich lipid stores^73,74^. For example, large calanoid copepods species (~ 3.5 mm) accumulate between 17 and 74% of lipids (dry mass^73^), and juveniles and adults of Antarctic krill are comprised of between 36 and 44% lipid, respectively (dry mass^74^). These substantial lipid stores undoubtedly render copepods and euphausiids as energetically valuable food sources for SRWs in the waters of Península Valdés. Thus, the present study suggests that SRWs make a trade-off choice to pursue these larger abundances and greater biomass, despite performing energetically expensive dives to the seafloor to access these high-quality prey. Moreover, this study provides baseline information for characterizing how this region is utilized by SRWs during their stay in Península Valdés. Protecting right whales requires a better understanding of their habitat use on finer spatial and temporal scales. Península Valdés has been identified as an important calving ground for this whale species; however, there is currently a consensus that SRWs feed during their stay in this area. Therefore, the information provided by this study may help natural resource managers in predicting right whale movements based on the concentration of calorically rich zooplankton prey within the region.

The data presented here constitute the first deployments of CRITTERCAMs on SRWs. While several studies have reported SRW foraging at Península Valdés mainly during spring months on copepods, which dominate the zooplankton community at surface and subsurface layers^13,60^, to date, no studies have attempted to assess the bottom prey of this whale species. Deployments have provided new knowledge on the underwater activities of SRWs at Península Valdés calving ground. In this study, CRITTERCAM video footage and zooplankton samples allowed us to know the association between SRW dives and the vertical distribution of zooplankton in the deeper waters of Golfo Nuevo. We observed that SRWs feeding underwater in concentrating zooplankton. Moreover, our observations revealed that the diving depth registered for SRWs were close to those of the seafloor where the whales were tagged, indicating that SRWs forage near the bottom when diving in Golfo Nuevo. Our findings complement those reported by D’Agostino et al.^17^, who observed SRWs surfacing with mud on their heads at the end of extended dives in Golfo Nuevo from mid to late spring, suggesting that SRWs feed in proximity to the seafloor. In this study we confirmed this behavior supported by data. However, in future studies, to understand the foraging dive behavior of SRWs will be necessary to deploy cameras equipped with high-resolution inertial sensors (e.g., triaxial accelerometers, magnetometers and gyroscopes), enabling the measurement of behavioral metrics (e.g., whale orientation, speed, heading, acceleration). The findings of this study contribute to our understanding of SRW feeding behaviors by demonstrating that they dive to exploit dense concentrations of large zooplankton aggregates in the deeper waters of Golfo Nuevo. The daily vertical migration of zooplankton influences the behavior and ecology of predators, such as baleen whales^42^. Copepods and euphausiids migrate downward out of the euphotic zone during the day to avoid being eaten by visual predators. However, this escape strategy makes them available for large whales during day, which employ a variety of sensory mechanisms to locate and capture prey at depths where little light is available. For instance, in baleen whales, it has been suggested that rostral vibrissae or sensory tubercles function as mechanoreceptors during feeding, aiding in the detection of water or prey movements as well as the location of zooplankton patches^75–77^. Murphy et al.^77^ further propose that right whales utilize their vibrissae to discern patch boundaries and evaluate densities within the patches, thereby optimizing their foraging efficiency and success.

Notably, deployments on adult females allowed us to observe the behavior of their calves. Three calves that were observed at the surface were also recorded near the seafloor, in close proximity to their mothers. While this behavior has been previously reported in calves of NARWs^59^, to the best of our knowledge, this is the first study to report that SRW calves dive to similar depths as adult and juvenile individuals. Baumgartner & Mate^59^ tagged NARW calves and found that they fed by diving to depths below 100 m. However, in this study, deployments were only on adult whales and juveniles; therefore, we cannot determine if the calves feed by diving to the bottom layer of Golfo Nuevo.

CRITTERCAM observations also revealed the vertical swimming speed. The highest speeds recorded throughout the entire dive cycle of SRWs occurred during the ascent. This speed difference between ascents and descents has been associated with the positive buoyancy of right whales^78^. During descents, whales use fluke strokes to counteract this buoyancy, but during ascents, they utilize it to power glides^78^. In addition, dive profiles showed that SRWs performed U‐ and V‐ shaped dives in Golfo Nuevo. Tagging studies that examined dive profiles of NARW feeding on copepods defined V-shaped dives as non-feeding, while identifying U-shaped dives as likely foraging^59,79^. However, the results presented here suggest that SRWs were feeding on zooplankton (copepods and euphausiids) at depth during both in U‐shaped and V-shaped dives. Video data from our SRW deployments demonstrated an increase in zooplankton abundance at depth in both dive types, indicating that feeding likely occurred in all our dive recordings. Moreover, we observed that SRWs moved their heads when prey densities increased both in U‐ and V-shaped dives, and as mentioned above, we interpreted those head movements as evidence that the SRW opened its mouth and feed during diving. This feeding behavior thorough descent and ascent phases (consistent with V-shaped dives) is in agrees with findings reported for euphausiids-feeding whales. For example, several studies have demonstrated that rorqual whales (Balaenopteridae) are characterized by rapid engulfment and subsequent filtration to optimize foraging efficiency, maximizing prey capture when they feed by diving on euphausiids with strong escape responses^20,21^. Nevertheless, to improve our understanding of the feeding behavior of SRWs, future studies should enhance the CRITTERCAM system to provide multi-directional viewing capabilities. Access to more concurrent views, such as those in front of and behind the whales simultaneously, would significantly improve the observation and understanding of underwater behaviors. Furthermore, incorporating low-light video recording capabilities into the CRITTERCAM would increase the ability to document whale behavior at greater depths and provide clearer imaging of their environment. In addition, integrating GPS recording and tracking abilities would allow for the recording of the whale’s track as it moves between locations. Likewise, the use of higher resolution cameras in CRITTERCAM recordings would enhance the quality of the data collected.

This study shows that in Golfo Nuevo, SRWs forage by diving on large calanoid copepods and euphausiids that aggregate in deeper waters. We observed that the zooplankton community at the bottom was mainly represented by large calanoid copepods (CIV), *C. australis* (CV and adult females), and *E. lucens* (FIV-FV and juvenile stages). *Calanus australis* is widely distributed in the inner and middle shelf waters of Argentina and is the most abundant calanoid species along the coast of southern Patagonia^80^. In this region, *C. australis* typically exhibits higher densities on the middle shelf within the upper 100 m^80^. In addition, this copepod is numerically important in mesozooplankton assemblages in both Golfo Nuevo and Golfo San José during mid-spring in superficial zooplankton samples^13,55^. Previous studies have reported that SRWs feed on *C. australis* at the surface in Golfo Nuevo^13,55^. Moreover, remains of *C. australis* -mainly CV- were found in fecal samples from SRWs collected at Península Valdés^7^. However, our study provides the first evidence that SRWs dive to feed on *C. australis*, which is highly concentrated at depths around 100 m. It is noteworthy at this point that the average body size found for adult females of *C. australis* at the bottom was significantly larger than the one near the subsurface. Our findings are in agreement with those of Baumgartner et al.^42^, who observed that the late stages of the calanoid copepod *Calanus finmarchicus* descending to deeper waters were larger and had a higher lipid content compared to those found in surface waters. These authors reported that well-fed copepods with larger oil sacs are more likely to perform DVM throughout the water column. Therefore, our study suggests that when SRWs feed at the bottom, they consume more calorically rich food. Furthermore, it is worth mentioning that while *C. australis* is known to exhibit DVM in the Argentine Sea^80^, neither their abundance at depth layers nor their vertical migration behavior in the gulfs off Península Valdés have been studied so far. In this sense, the present study is the first to demonstrate that *C. australis* is highly distributed at the bottom of the water column during daylight hours and probably performs DVM in Golfo Nuevo.

*Euphausia lucens* is an abundant euphausiid species widely distributed in the Argentine Sea^64,81^. However, this species is sporadically found and occurs in low abundance in zooplankton samples from shallow (≤ 30 m) and intermediate (~ 70 m) layers collected during the daytime in the gulfs off Península Valdés^13,60^. According to our data, DVM might explain the low density of *E. lucens* found in samples collected in previous studies in Golfo Nuevo^13,60^. It has been demonstrated that both juvenile and adult stages of *E. lucens* migrate extensively throughout the water column^82^ and remained at or near the bottom during daylight hours and ascending toward the surface at night as shown for the Golfo San Jorge in Argentinean Patagonia^45^. Here, the highest abundance of *E. lucens* in older stages was found aggregated near the bottom. However, the sampling methodology likely led to an underestimation of euphausiids as a consequence of their strong swimming speeds and ability to avoid net tows of short duration at low speed used here as sampling method^83,84^. As a result, although the recorded abundances of euphausiids were high, the registered density may be underestimated concerning the actual abundance at the bottom layer, as could be the case when studying it with other methodologies such as echosounders^45^. Additionally, we recognize that our sampling system’s sled shape resulted in the retention of numerous specimens in the net rather than in the collector, possibly affecting the accurate representation of the abundance of larger zooplankton species at the bottom. Despite this limitation, our study highlighted higher euphausiid abundances at the bottom compared to the subsurface, providing insight into the foraging preferences of SRWs regarding prey quality and quantity during dives in Golfo Nuevo.

## Conclusion

This study is the first to demonstrate that SRWs forage in deeper water layers on large, calorically rich prey. To date no studies have attempted to assess the dive behavior of SRWs along with the distribution of their prey. Therefore, our findings offer new insights into the foraging ecology of SRWs, contributing to a better understanding of the relationship between SRWs and their prey. This study demonstrates that SRWs efficiently exploit aggregations of high-energy prey during the day when large zooplankton organisms are aggregated in deeper layers. Another new finding of this study is that calves at the calving ground of SRWs in Península Valdés dive to similar depths as adult and juvenile individuals. With this information as a baseline, we suggest future studies should replicate this investigation throughout the whale season (June-December) to determine when whales are most likely to engage in feeding dives in Golfo Nuevo and to identify their prey in deep waters during their stay in this gulf. Additionally, based on our results, we highlight the importance of deploying CRITTERCAMs on whale calves to understand their underwater activities.

The present study reports, for the first time, the vertical distribution of large zooplankton in Golfo Nuevo. Our findings reveal that large calanid copepods and euphausiids dominate the zooplankton community and represent a significant portion of the zooplankton biomass at the bottom. Therefore, this study provides initial evidence suggesting that larger zooplanktonic organisms undertake DVM in Golfo Nuevo. Given the pivotal role of DVM in marine ecosystem processes (e.g., predator–prey interactions, population dynamics, and contributions to biogeochemical processes such as the transport of dissolved inorganic carbon and nitrogen to deep water^85^), future investigations and greater efforts should be made to understand the pelagic zooplankton ecology in the waters of Península Valdés.

Finally, our study reinforces the importance of Península Valdés as a multiple-use area for SRWs. Therefore, it is crucial that management policies focus not only on charismatic species like marine mammals but also on all components of the ecosystem, particularly emphasizing the components at the base of food webs.

### Supplementary Information

Supplementary Information.

Supplementary Video 1.

Supplementary Video 2.

Supplementary Video 3.

Supplementary Video 4a.

Supplementary Video 4b.

Supplementary Video 5.

Supplementary Video 6.
