# Supplementary material for: Foraging dives of southern right whales (Eubalaena australis) in relation to larger zooplankton size prey availability in Golfo Nuevo, Península Valdés, Argentina
Source: Sci Rep. 2024 Jun 20;14:14211. doi: 10.1038/s41598-024-63879-y (PMC11190224; doi:10.1038/s41598-024-63879-y)
Supplement: Supplementary file 1 — Supplementary Information. [file 41598_2024_63879_MOESM1_ESM.pdf]

ONLINE RESOURCES

Article: Foraging dives of southern right whales (*Eubalaena australis*) in relation to larger zooplankton size prey availability in Golfo Nuevo, Península Valdés, Argentina

Journal: Scientific Reports

Authors: Valeria C. D’Agostino, Ariadna C. Nocera, Kyler Abernathy, Alex Muñoz Wilson, Mariano A. Coscarella, Mariana Degrati

Corresponding authors: Centro para el Estudio de Sistemas Marinos (CESIMAR), CCT CENPAT, CONICET, Blvd. Brown 2915, U9120ACV Puerto Madryn, Chubut, Argentina. E-mail: dagostino@cenpat-conicet.gob.ar (V. C. D’Agostino) or Email: degrati@cenpat-conicet.gob.ar (M. Degrati)

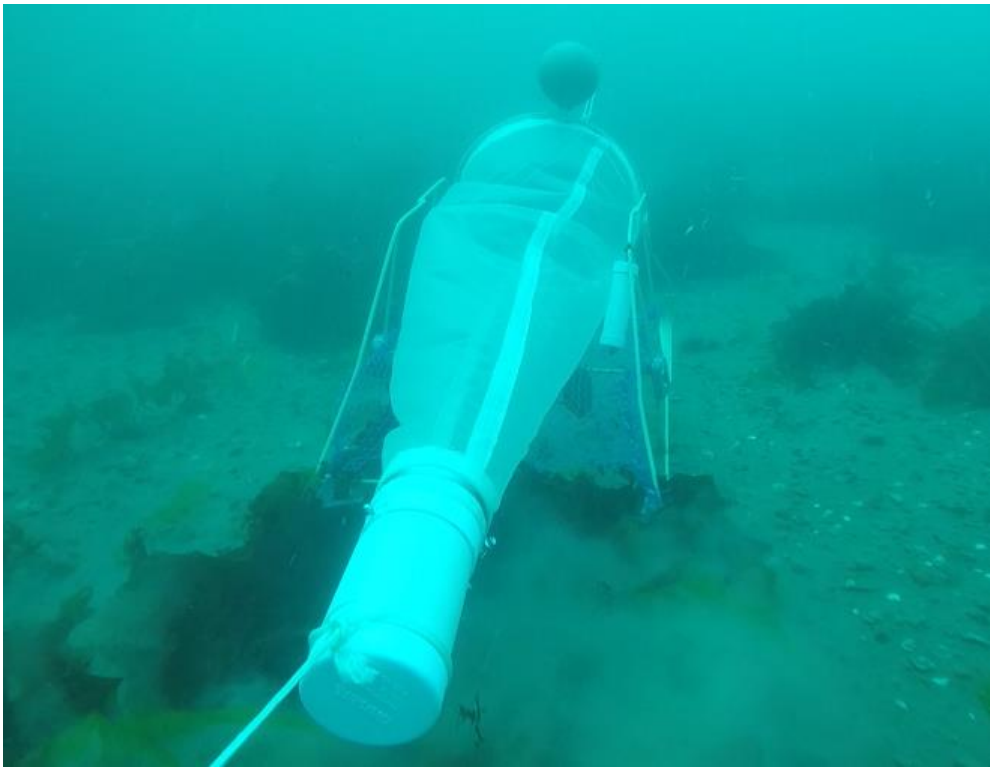

Figure S1: The sled designed for the bottom-depth tow equipped with a plankton net (335 µm mesh, 50 cm mouth diameter), a mechanical flowmeter at the net mouth, and temperature and pressure sensors.

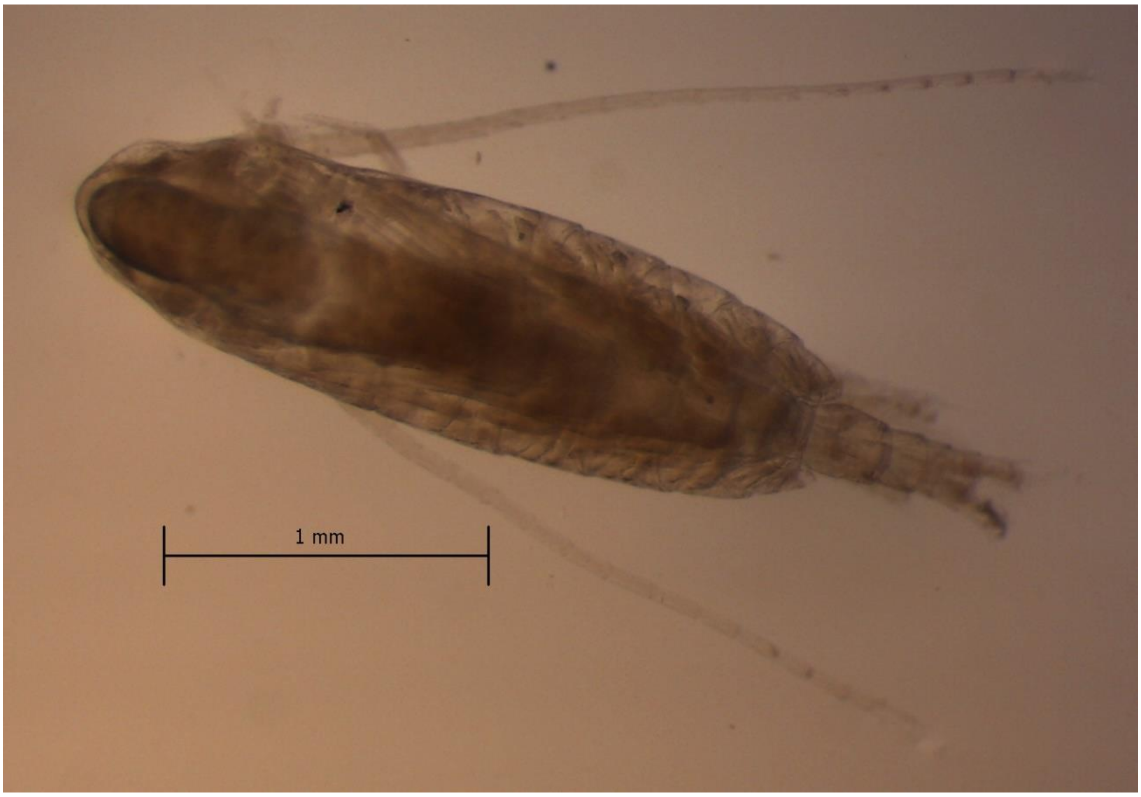

Figure S2: *Calanus australis* adult female.

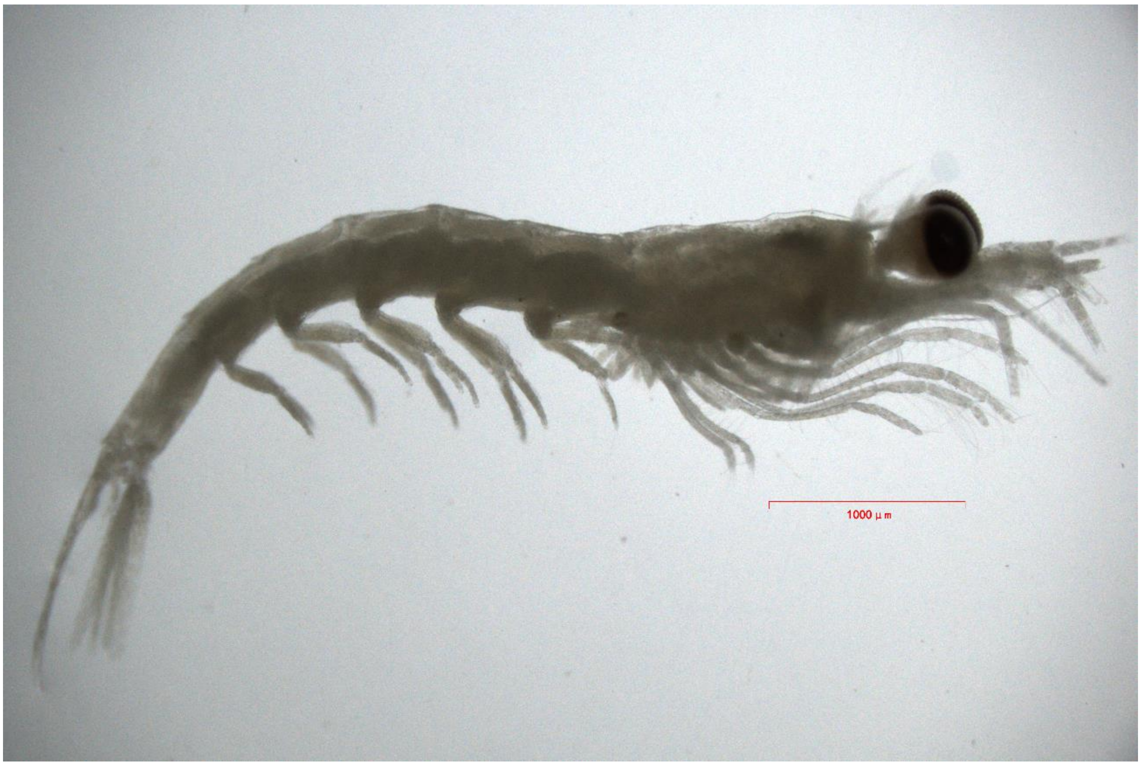

Figure S3: *Euphausia lucens* juvenile.

Video S1: CRITTERCAM deployment on southern right whale (SRW, *Eubalaena australis*) in Golfo Nuevo, Península Valdés, Argentina. Video credits: Samuel Deleon and Steve Spence, National Geographic Pristine Seas.

Video S2: Edited video from southern right whale (SRW, *Eubalaena australis*) deployment ID1 (adult female). Note superficial (3 m) feeding behaviour of adult female with CRITTERCAM alongside another SRW individual at 35 s, both with their mouths open. At 46 s, the descent phase begins, note the increase in large particle density and speed (likely large copepods and euphausiids), calf visible at ~100 m. Subsequently, ID1 continues feeding at the surface.

Video S3: Edited video from southern right whale (SRW, *Eubalaena australis*) deployment ID2 (adult female). Calf near its mother during the descent phase and at the bottom when the mother reached her maximum depth of 84 m. Note the increase in large particle density and speed (likely large copepods and euphausiids).

Video S4a: Edited video from southern right whale (SRW, *Eubalaena australis*) deployment ID4 (juvenile). Note the high density of large particles (likely large copepods and euphausiids) while the whale diving feeding at ~ 100 m.

Video S4b: Edited video from southern right whale (SRW, *Eubalaena australis*) deployment ID4 (juvenile). Note the high density of particles while the whale diving feeding at ~ 40 m.

Video S5: Edited video from southern right whale (SRW, *Eubalaena australis*) deployment ID8 (juvenile). Note the movement of its head when prey densities increased, as well as the high density of large particles (likely large copepods and euphausiids) while the whale diving feeding at ~ 115 m.

Video S6: Edited video from southern right whale (SRW, *Eubalaena australis*) deployment ID10 (adult female). Note the movement of her head as well as the high density of large particles (likely large copepods and euphausiids) while she was feeding at ~ 75 m. Calf visible at ~73 m (at 11 s).
